# Validity of the “Drift without pronation” sign in conversion disorder

**DOI:** 10.1186/1471-2377-13-31

**Published:** 2013-04-01

**Authors:** Corinna Daum, Selma Aybek

**Affiliations:** 1 Service de Neurologie, Clinical Neurosciences Department, Lausanne University Hospital (CHUV), Lausanne, Switzerland

**Keywords:** Conversion disorder, Pronator drift, Arm paresis, Functional symptom

## Abstract

**Background:**

Conversion disorder (CD) is a psychiatric disorder, yet the diagnosis cannot be established without the expertise of a neurologist, as distinguishing a functional from an organic symptom relies on careful bedside examination. Joseph Babinski considered the absence of pronator drift as a ‘positive sign’ for hysterical paresis but the validity of this sign has never been evaluated. The aim of this study was to examine the sensitivity and specificity of the “drift without pronation” sign.

**Methods:**

Twenty-six patients with unilateral functional upper limb paresis diagnosed with CD (DSM-IV) and a control group of 28 patients with an organic neurological condition were consecutively included. The arm stabilisation test was performed with arms stretched out in full supination, fingers adducted, eyes closed for 10 seconds. A positive “drift without pronation” sign was defined by the presence of a downward drift without pronation.

**Results:**

All CD subjects (100%) displayed a positive sign when only 7.1% of organic subjects did (Fisher’s p < 0.001). The sign yielded a sensitivity of 100% (95% CI:84%-100%) and a specificity of 93% (95% CI:76%-98%).

**Conclusion:**

The observation of a “drift without pronation” sign is specific for Conversion Disorder and can be of help in making a quick distinction between organic and functional paresis at the bedside.

## Background

Conversion disorder (CD) is a psychiatric disease but the diagnosis cannot be established without the help of a trained neurologist, as the main criterion formulates that the neurological symptom “cannot be explained by a neurological or general medical condition”. The crucial issue for neurologists is thus to reliably distinguish such functional or ‘medically unexplained’ symptom from an organic ‘medically explained’ one. In order to do so, clinicians look for inconsistencies at the neurological examination and rely on the presence of ‘positive signs’, known to be commonly found in functional symptoms [1]. This is especially important, as a functional and an organic disorder can co-exist in a single patient and are known to represent “functional overlays” [2] in neurological conditions. In this context, ‘positive signs’ play a major role and establishing their sensitivity and specificity has been a recent focus of clinical research. While functional lower limb paresis can now reliably be diagnosed with the help of the Hoover’s sign [3] and the abductor sign [4], few signs are available for upper limb paresis [5]. During the arm stabilisation test, the presence of a pronator drift, a mild elbow flexion and passive abduction of the little finger and sometimes adjacent fingers (Souques’ sign) are understood as signs of upper motor neuron dysfunction. Babinski described ‘absent pronator drift’ as a sign of hysterical paresis [6]. Observing a weak arm drifting downward as a whole rather than in a pronation movement [6,7] has thus been seen as a functional ‘positive sign’ but its clinical value has never been formally tested to the best of our knowledge.

We set out to carefully examine the arm stabilisation test in a prospective controlled study, and to look for the presence of a drift with and without pronation in order to estimate the sensitivity and specificity of the “drift without pronation” sign.

## Methods

### Subjects

Twenty-six patients with an established diagnosis of Conversion Disorder (according to DSM-IV-TR) and a control group of twenty-eight patients with an organic lesion (ischemic, inflammatory, infectious or tumoral) presenting unilateral upper limb weakness were consecutively included between 01.03.2011 and 01.03.2012 at our in- and out-patients University tertiary care Neurological Department. Upper limb weakness was defined by a National Institute of Health Stroke Scale (NIHSS) of ≥1 (drift within 10 seconds) for the affected limb and/or an objective paresis on the Medical Research Council (MRC) Scale for Muscle Strength (<5/5) in shoulder abduction, elbow flexion and wrist extension. Exclusion criteria were complete arm paralysis (NIHSS score 4 and MRC scale 0/5 for all 3 muscle groups), age of <18 and >85 years old, severe aphasia, dementia and acute confusional state. All patients had a detailed neurological examination, brain imaging (CT and/or MRI) and if necessary other evaluations (electroneuromyography, lumbar puncture, spine MRI, others) to confirm/exclude an organic lesion. All subjects with Conversion Disorder were assessed by a trained Liaison psychiatrist to confirm the diagnosis. All subjects gave their written informed consent and the study was approved by the local ethics committee (Commission cantonale d’éthique de la recherché sur l’être humain, Université de Lausanne, protocole 03/11).

### Procedure

All subjects were examined by one of the two authors with the arm stabilisation test. Subjects were sitting in front of the examiner with arms stretched out, palms up in a full supination position, fingers adducted, eyes closed for 10 seconds, instructed to keep that position as long as possible. During the downward drift, we carefully looked for a pronation movement. When even a slight pronation was observed it was judged as being present. In cases of rapid drift due to severe paresis (NIHSS = 3), as the downward fall of the limb was too rapid for the examiner to identify a pronation movement, we looked for the final position of the hand (which dropped on the patient’s knee) either in a pronated or supinated position. We defined a positive sign of “drift without pronation”, when the arm was seen to go down and no pronation occurred during the descent and when the final hand position was in supination. The sign was considered negative when the arm was seen to go down and a pronation was present and when the final hand position was in pronation.

We compared the frequency of this sign between the two groups using the two-tailed Fisher’s exact test and calculated its sensitivity and specificity, towards a diagnosis of Conversion Disorder.

## Results

### Demographics

The 26 Conversion Disorder patients (17 female, mean age 41.1±10.2 years) consisted of 11 right-sided and 15 left-sided upper limb weaknesses. Eighty-five percent of patients (22/26) had sensorimotor hemisyndrome and 15% (4/26) had pure motor hemisyndrome. Fifty-four percent (14/26) had a brachio-crural deficit and 46% (12/26) a facio-brachio-crural involvement.

The 28 organic patients (14 female, mean age of 62.9±14.7 years) consisted of 13 right-sided and 15 left-sided weaknesses. Seventy-one percent of patients (20/28) had sensorimotor hemisyndrome and 29% (8/28) had a pure motor hemisyndrome. Fourteen percent (4/28) had a brachio-crural deficit, 68% (19/28) a facio-brachio-crural, 14% (4/28) a facio-brachial and 4% (1/28) an isolated upper limb deficit. Twenty-three organic patients suffered from Stroke, 2 from Multiple Sclerosis, 1 from Progressive Multifocal Leukoencephalopathy, 1 from cerebral lymphoma, 1 from cerebral toxoplasmosis.

### The arm stabilisation test

All 26 Conversion Disorder patients (100%) had a positive “drift without pronation” sign when only two organic patients (7.1%) displayed a positive sign (p < 0.001).

The sensitivity of the test was 100% (95% CI:84%-100%) and the specificity 93% (95% CI:76%-98%).

## Discussion

This prospective controlled study indicates that the bedside observation of a “drift without pronation” is a useful and reliable clinical sign to discriminate between functional and organic upper limb weakness. This ‘positive sign’ was observed in all (100%) Conversion Disorder subjects and in only 7.1% of organic patients. When looking for ‘positive signs’ of functional deficits, clinicians are interested in having highly specific tests, minimizing false-positive results. Our study revealed a specificity of 93%, suggesting that this test is indeed helpful in clinical practice to identify a functional disorder.

Pronator drift is understood as a sign of upper motor neuron disorder and is considered as an indicator of a structural cerebral lesion [8-11] in subjects with moderate paresis. Of note, this sign is usually considered positive when either a drift or a pronation is observed [8,9,12] and only one study [10] carefully looked at both aspects separately, finding that amongst 38 patients with a pronator drift sign, 74% had both a downward drift and a pronation, when 26% had an isolated pronation. Our findings suggest that when performing the stabilisation arm test, one should carefully look at both components of the pronator drift; the presence of a pronation will favour an organic cause, either in isolation or with a downward drift, whereas the observation of a drift *without* pronation will be highly suggestive of a functional paresis.

Our study has some limitations. The examiners where not blinded to the subjects’ diagnoses, the interpretation of a ‘positive sign’ could have been biased. In order to minimize the influence of such personal subjective judgement, the evaluation was dichotomised (present or absent) and even a slight pronation movement was reported as present. The interobserver reliability of bedside neurological signs of hemiparesis [13], Barré sign (downward drift) [14] and pronator drift with fingers adducted [15] has however been reported as good (Kappa scores ranging from 0.55 to 0.77) so it can reasonably be expected that our assessments were reliable, even though only a blinded design including an independent rater could confirm it.

As no gold standard to diagnose functional weakness exist, there is a potential risk for circular reasoning bias: if the studied sign is also used in the diagnosis process, the reported specificity and sensitivity are overestimated. We tried to minimize this bias by strictly using the DSM-IV criteria to establish the diagnosis of conversion disorder without specifically using the “drift without pronation”. Moreover, the diagnosis and the testing were not performed by the same doctor.

## Conclusion

The use of the “drift without pronation” sign can be recommended, as it was found in this unblinded study to be highly specific for functional upper limb paresis. However, further studies, including this sign and combining it with other functional signs in a blinded design, will help better refine this crucial issue of distinguishing functional from organic symptoms at the bedside; as for now only clinical criteria are used to diagnose functional deficits in Conversion disorder, this will lead to an improvement in the care of these patients.

## Competing interest

The authors have no competing interest and nothing to disclose.

## Authors’ contributions

Both authors, SA and CD, included the patients, performed the test, ran the statistical analyses. CD wrote the first draft of the manuscript and SA critically reviewed and edited it. Both authors read and approved the final manuscript.

## Pre-publication history

The pre-publication history for this paper can be accessed here:

http://www.biomedcentral.com/1471-2377/13/31/prepub
